# Three-dimensional Imaging Coupled with Topological Quantification Uncovers Retinal Vascular Plexuses Undergoing Obliteration

**DOI:** 10.7150/thno.53073

**Published:** 2021-01-01

**Authors:** Chih-Chiang Chang, Alison Chu, Scott Meyer, Yichen Ding, Michel M. Sun, Parinaz Abiri, Kyung In Baek, Varun Gudapati, Xili Ding, Pierre Guihard, Kristina I. Bostrom, Song Li, Lynn K. Gordon, Jie J. Zheng, Tzung K. Hsiai

**Affiliations:** 1 Department of Bioengineering, University of California, Los Angeles, Los Angeles, CA.; 2 Division of Neonatology and Developmental Biology, Department of Pediatrics, David Geffen School of Medicine at UCLA, Los Angeles, CA.; 3 Department of Medicine, David Geffen School of Medicine at UCLA, Los Angeles, CA.; 4 Department of Ophthalmology, Stein Eye Institute, David Geffen School of Medicine at UCLA, Los Angeles, CA.; 5 Greater Los Angeles VA Healthcare System, Los Angeles, CA.; 6 Medical Engineering, California Institute of Technology, Pasadena, CA.

**Keywords:** Light-sheet fluorescence microscopy, Primary and secondary plexus, Vertical sprouts, Oxygen-induced retinopathy, Retinal vasculature

## Abstract

**Introduction:** Murine models provide microvascular insights into the 3-D network disarray seen in retinopathy and cardiovascular diseases. Light-sheet fluorescence microscopy (LSFM) has emerged to capture retinal vasculature in 3-D, allowing for assessment of the progression of retinopathy and the potential to screen new therapeutic targets in mice. We hereby coupled LSFM, also known as selective plane illumination microscopy, with topological quantification, to characterize the retinal vascular plexuses undergoing preferential obliteration.

**Method and Result:** In postnatal mice, we revealed the 3-D retinal microvascular network in which the vertical sprouts bridge the primary (inner) and secondary (outer) plexuses, whereas, in an oxygen-induced retinopathy (OIR) mouse model, we demonstrated preferential obliteration of the secondary plexus and bridging vessels with a relatively unscathed primary plexus. Using clustering coefficients and Euler numbers, we computed the local versus global vascular connectivity. While local connectivity was preserved (*p* > 0.05, n = 5 vs. normoxia), the global vascular connectivity in hyperoxia-exposed retinas was significantly reduced (*p* < 0.05, n = 5 vs. normoxia). Applying principal component analysis (PCA) for auto-segmentation of the vertical sprouts, we corroborated the obliteration of the vertical sprouts bridging the secondary plexuses, as evidenced by impaired vascular branching and connectivity, and reduction in vessel volumes and lengths (*p* < 0.05, n = 5 vs. normoxia).

**Conclusion:** Coupling 3-D LSFM with topological quantification uncovered the retinal vasculature undergoing hyperoxia-induced obliteration from the secondary (outer) plexus to the vertical sprouts. The use of clustering coefficients, Euler's number, and PCA provided new network insights into OIR-associated vascular obliteration, with translational significance for investigating therapeutic interventions to prevent visual impairment.

## Introduction

Aberrant retinal angiogenesis is a hallmark of numerous retinal disorder-mediated vasculopathies, including retinopathy of prematurity (ROP) and diabetic retinopathy (DR), resulting in visual impairment 1, 2. Premature newborns are susceptible to hyperoxic injury and the development of retinopathy of prematurity. They often require supplemental oxygen therapy due to the immature development of their lungs, and this hyperoxia exposure leads to attenuation of the retinal vascular bed 3-5. Subsequently, these newborns further develop ischemia-induced neovascularization in the retina due to local hypoxia and increased metabolic demand 4, 6. Similarly, diabetic patients are also prone to developing proliferative retinopathy and aberrant neovascularization as a result of hypoxia 7, 8.

To quantify the microvascular damage that occurs early in DR, ophthalmologists have relied on several clinical imaging modalities, including fundus photography, fluorescein angiography (FA), and optical coherence tomography angiography (OCTA). These imaging modalities allow for screening and monitoring of vision-threatening complications in adults 9, 10. OCT-angiography enables identification of preclinical retinal vascular changes, including the remodeling of the foveal avascular zone (FAZ), capillary nonperfusion, and reduction of capillary density 11-15. However, the quality of the images obtained using these modalities in neonates and young children is often limited by ocular movement and a restricted field of view.

To elucidate the mechanisms underlying vascular abnormalities seen in the presence of ROP, researchers have utilized mouse models of oxygen-induced retinopathy (OIR) 16, 17. Akin to early ocular development in humans, the postnatal mouse retina is considered a viable model to underpin the development of retinal vascular network 1, 2, 16. The current gold standard to image and quantify changes in the murine retinal vascular network typically utilizes whole-mount samples with 2-D analysis 17-21. Previous groups have utilized 3-D imaging to demonstrate vaso-obliteration in the rat model 22 and report the local 'knotted' morphology and vascular tufts during neovascularization in murine OIR 23. To quantify the early abnormalities and progression of OIR in 3-D for the entire retinal vasculature, we hereby coupled selective plane illumination microscopy also known as light-sheet fluorescence microscopy (LSFM), with quantitative 3-D topological analyses to interrogate the entire retinal vasculature in response to hyperoxia-induced microvascular obliteration 24-28. Unlike confocal or two-photon microscopy commonly applying a point scanning approach, LSFM generates a sheet of laser light to rapidly scan across optically-cleared specimens 24, 29. This approach provides an entry point to investigate the murine ocular system in 3-D with rapid imaging acquisition to minimize photo-bleaching. LSFM further provides the high axial resolution needed to quantify the multi-layered vascular network; namely, the vertical sprouts embedded between the primary and secondary plexuses 1, 16, 30.

To this end, we optimized an optical clearing technique to preserve the 3-D conformation of the hemispherical murine retina and its microvasculature for LSFM imaging. Following hyperoxia exposure (75% O_2_) to post-natal mice for 5 days (P7-P12), we quantified the spatial variations in vascular obliteration and observed a preferential loss of the vertical sprouts and secondary plexuses at day P12. Using principal component analysis (PCA), we developed an automated segmentation algorithm to demonstrate a significant reduction in the volume fraction of the vertical sprouts that bridge the primary and secondary plexuses. In oxygen-induced retinopathy (OIR), we calculated both Euler numbers and clustering coefficients to reveal a reduction in the global vascular connectivity, but a preserved local connectivity. Our topological analysis of the large data set provided by light-sheet imaging, including PCA, further corroborated the deep capillary obliteration following oxygen-mediated retinopathy. We hereby establish topological quantification to unravel spatial variations in microvascular obliteration that preferentially affect the secondary plexus and vertical sprouts in murine OIR, allowing for the detection of early changes in hyperoxia-mediated microvascular disarray.

## Results

### Schematic pipeline for the 3-D quantitative analysis of murine retinal vasculature

**Figure 1:**
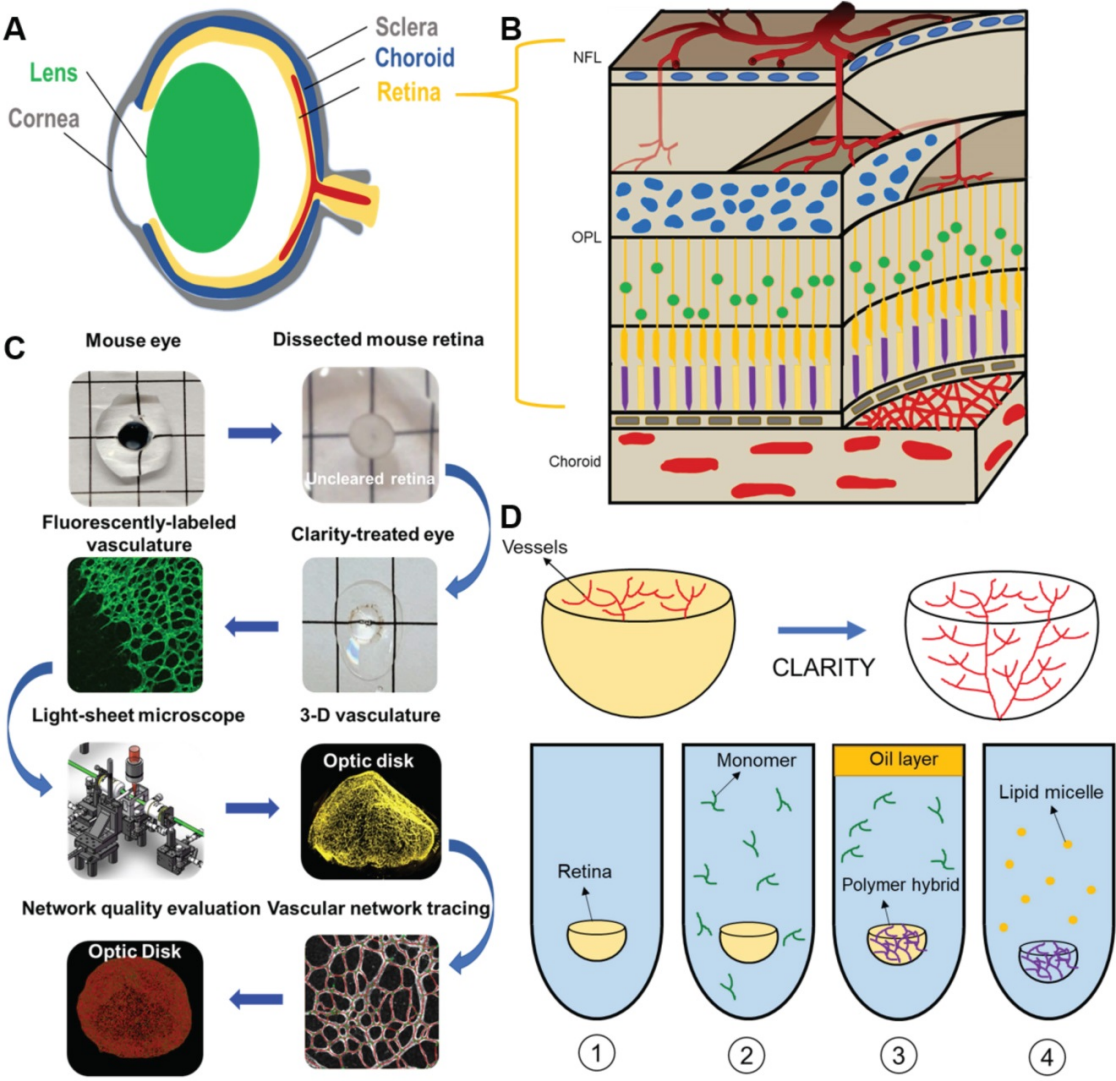
** Light-sheet fluorescence microscopy (LSFM) to uncover the 3-D microvascular network. (A)** A schematic illustration of the intact ocular globe, consisting of retinal and choroidal vasculature that were imaged and analyzed. **(B)** A superficial primary retinal vascular plexus lies in the nerve fiber layer (NFL), whereas the secondary plexus is located deep in the outer plexiform layer (OPL). **(C)** The pipeline to quantitatively analyze the 3-D hemispherical retinal vascular plexus using a optimized CLARITY method and LSFM. **(D)** An optimized passive CLARITY method was applied to optically clear the retina as articulated in the Methods section.

To achieve 3-D retinal microvasculature imaging and analysis (Figure 1A-B), we developed an eight-step pipeline for light-sheet imaging and quantification in the ensuing sections (Figure 1C). After euthanizing the mice at postnatal day 12 (P12), the ocular globes were enucleated and dissected to obtain the unscathed hemispherical retinas, and polymerization and lipid removal were performed in the monomer and clearing solution to achieve optical transparency (Figure 1D). Next, immunofluorescence staining with Isolectin B4 (Vector Lab, CA) was performed to label the vascular endothelium in preparation for light-sheet imaging. Following filament tracing, the morphology and topology of the retinal vasculature were characterized by quantifying the vascular branching points, Euler characteristics, and clustering coefficients.

### 3-D characterization of the vascular network under normoxic conditions

**Figure 2:**
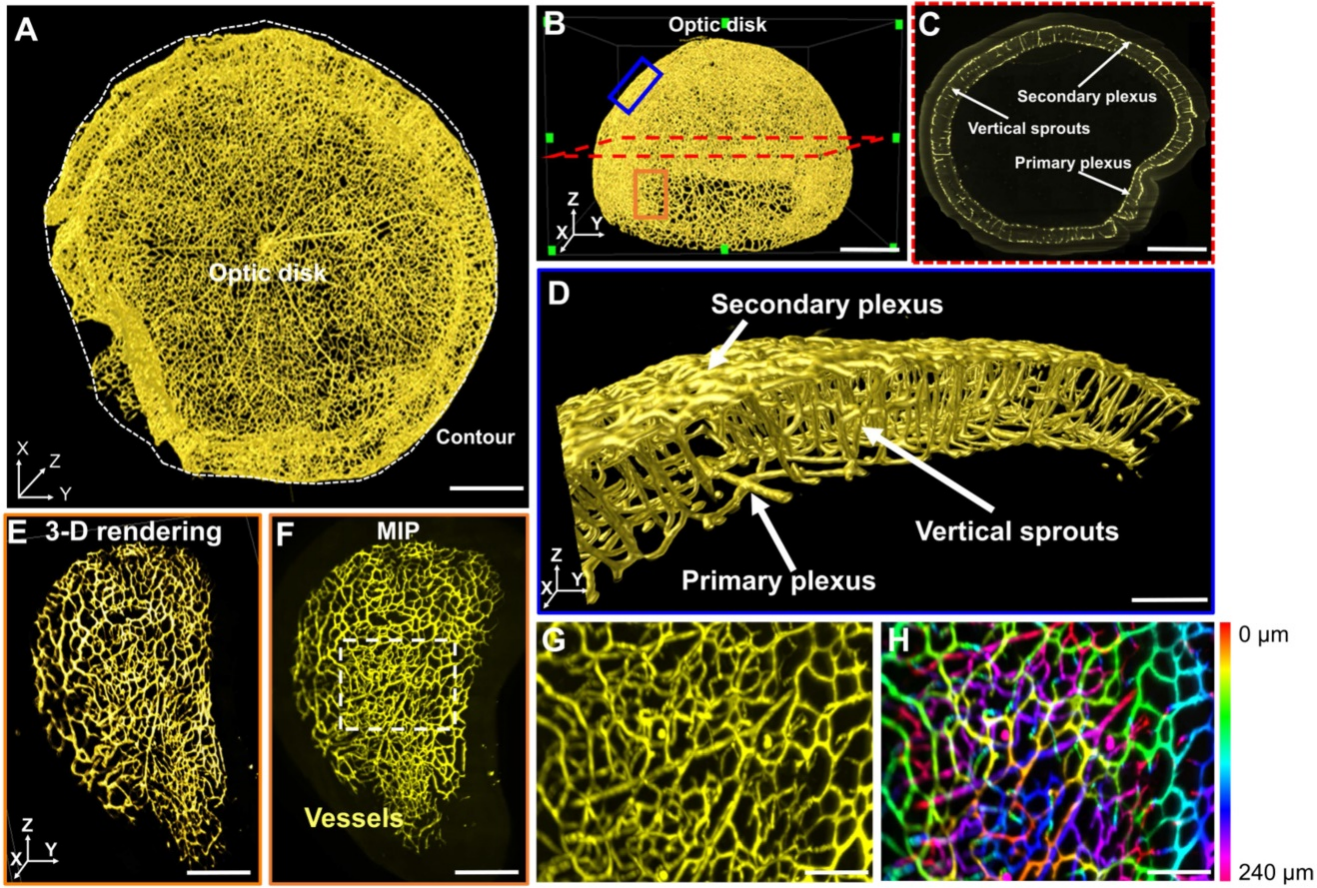
** LSFM imaging of the unscathed 3-D hemispherical retina. (A-B)** The unscathed vasculature in the 3-D retina was used to define representative regions of interest. The white dashed line depicts the contour of the hemispherical retina. **(C)** The vertical sprouts, bridging the primary and secondary plexus, are highlighted in the representative 2-D section (red dashed lines in **B**) of the retinal vascular network. **(D)** 3-D vertical sprouts between the primary and secondary vascular plexus (blue box in **B**) are located in the nerve fiber layer and the outer plexiform layer.** (E)** The 3-D and **(F)** 2-D peripheral regions (orange box in **B**) of the retina are enlarged. **(G)** The maximum intensity projection and **(H)** depth color-coded images of the capillary network (dashed box in **F**). Scale bars: (A-C) 500 µm; (D) 100 µm; (E-F) 300 µm; (G-H) 50 µm. Depth color-coded scale: 0~240 µm in (H).

LSFM was employed to unravel the 3-D vascular network in the hemispherical retina with endothelial cells labeled with Alexa Fluor 488 (Figure 2A-B). Representative volumes of interest (VOIs) were selected, including a 2-D section (red dashed line), peripheral vascular networks in the peripheral region (orange box), the middle region (blue box) (Figure 2B), and a 2-D slice (Figure 2C), to highlight the two-layered plexuses with 2-D connections. The volumetric rendering of the VOI (blue box) revealed the vertical sprouts bridging the primary and secondary plexuses in 3-D (Figure 2D, Movie S1). Light-sheet imaging also allowed for visualization of the 3-D vascular network from selected VOIs at various depths (Figure 2E-F), uncovering the interconnected capillary vascular bed as indicated by the color-coded depth imaging (Figure 2G-H). Taken together, the 3-D LSFM revealed the hemispherical vascular network to uncover the multi-layered capillary plexuses under normoxic conditions.

### Morphological and topological quantification of the 3-D retinal vascular network

**Figure 3:**
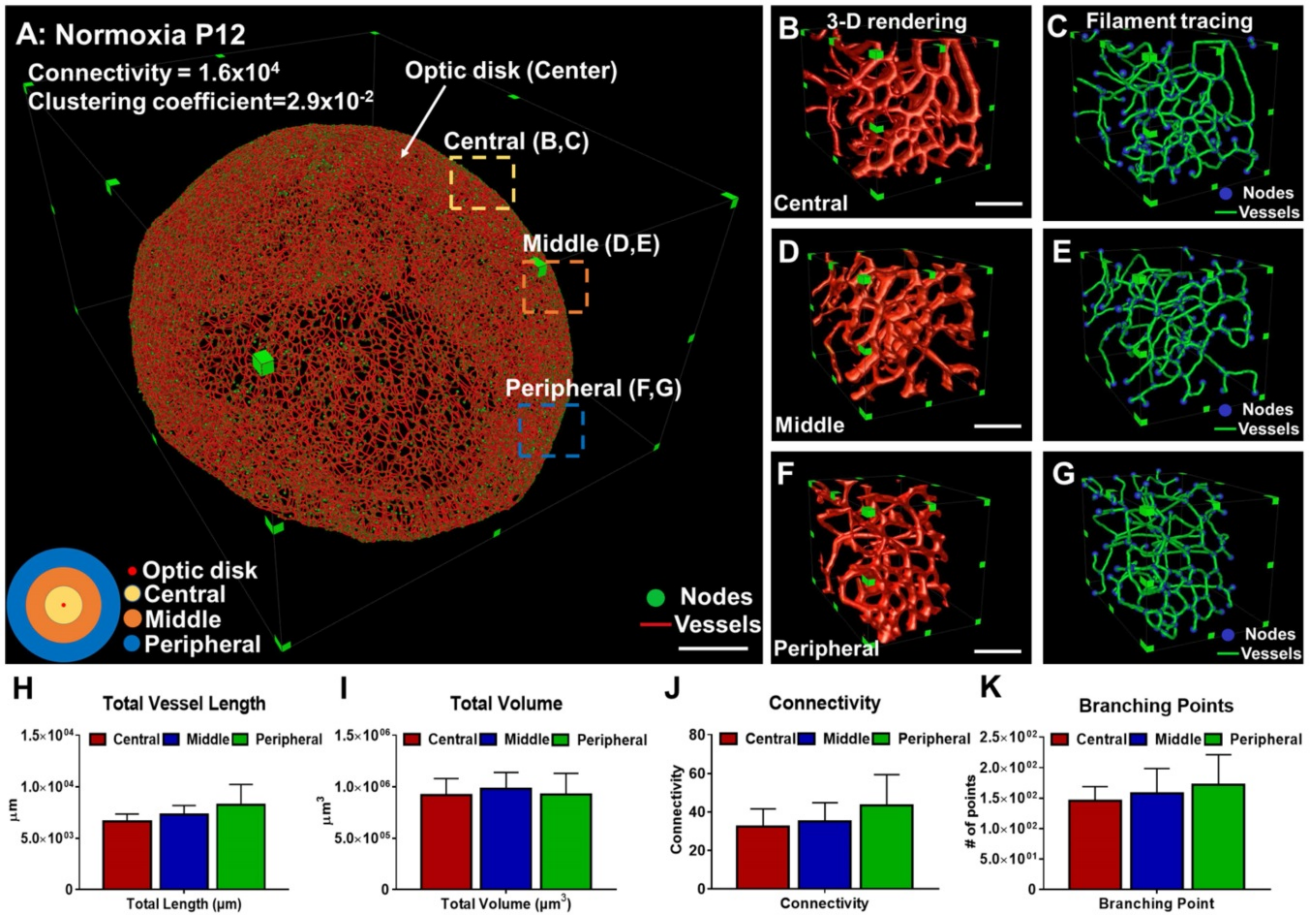
** Quantitative comparison between the regions of a healthy vascular network in P12 mice. (A)** 3-D filament tracing of retinal vasculature was performed from the unscathed hemisphere retina under normoxic conditions. Representative 3-D rendering **(B, D, F)** and filament tracing **(C, E, G)** of different volumes of interest (VOI) were demonstrated in the central (B, C), middle (D, E), and peripheral (F, G) regions of the unscathed retina. **(H-K)** Quantification of the morphological and topological parameters: vessel lengths, vessel volumes, connectivities, and branching points for different VOIs from the retina, demonstrating the capability and flexibility of our pipeline to quantify specific regions of interest (*p*>0.05 using one-way ANOVA with Tukey post hoc analysis, n = 5 per each region). Scale bar: (A) 500 µm; (B-G) 100 µm.

We applied the filament tracing strategy to characterize retinal morphology and topology. Vascular connectivity and clustering coefficients were quantified (Figure 3A, Movie S2), as illustrated by the interconnected nodes (in green) within the 3-D vascular network (in red). Clustering coefficients represent the degree of regional vascular connectivity, ranging from 0 to 1. The connectivity and mean clustering coefficients for the unscathed retina were 1.6×10^4^ and 2.9×10^-2^, respectively. The former coefficient indicates a large number of loops in the retinal vasculature, and the latter shows the low degree of clustering of these individual nodes. We interrogated the central (yellow disk), middle (orange rings), and peripheral (blue rings) regions of the hemispherical network (Figure 3A). We selected 5 volumes of interest (VOIs) from each of the regions as a standard assessment for retinopathy 10. The 3-D vascular network was visualized (Figure 3B, D, and F) and filament tracing was applied to characterize the vessel lengths, branching points, and connections in the central, middle and peripheral regions. Nodes were labeled in blue and segments in green (Figure 3C, E, and G). The means of the entire vessel lengths were 6.62×10^3^ ± 7.4×10^2^ µm for the central, 7.29×10^3^ ± 8.9×10^2^ µm for the middle, and 8.26×10^2^ ±1.9×10^3^ µm for the peripheral regions. In the absence of hyperoxia exposure, the differences among these 3 regions were statistically insignificant (*p* > 0.05, n = 5/region). The means of the entire vessel volumes were 9.19×10^5^ ±1.63×10^5^ µm^3^, 9.81×10^5^ ±1.59×10^5^ µm^3^, and 9.27×10^5^ ±2.5×10^5^ µm^3^, respectively. There were 146 ± 23.1 nodes and 32.3 ± 9.3 in connectivity in the central region, 157.3 ± 41.3 nodes and 35 ± 9.85 in connectivity in the middle region, and 172.3 ± 49.1 nodes and 43.3 ± 16.1 in connectivity in the peripheral region (Figure 3H-K). Thus, the differences in vascular lengths, volumes, connections, and branching points among the three representative regions of the retinal vasculature were statistically insignificant under the normoxic conditions.

### Changes in the morphological and topological measures in OIR

**Figure 4:**
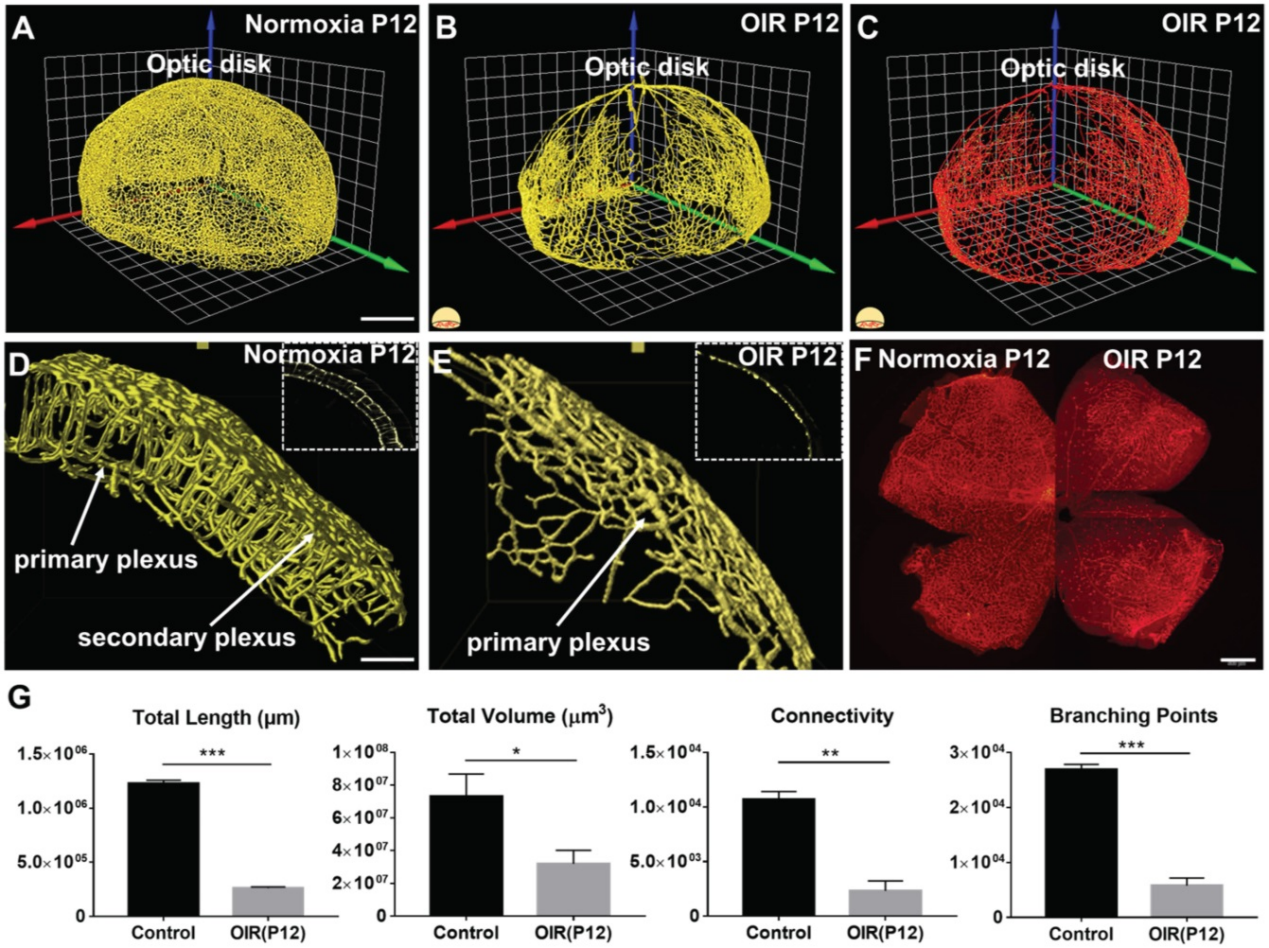
** The 3-D vascular network highlights the spatial variations in microvascular obliteration that occurred in the secondary plexus and vertical sprouts following hyperoxia-induced injury in P12 mice.** 3-D rendering of the retinal vasculature was performed from the **(A)** normoxia and **(B)** OIR intact retinas, revealing statistically significant obliteration of microvasculature in the OIR retina in P12 mice. **(C)** 3-D filament tracing of the vasculature was performed in the OIR group. **(D-E)** The results of 3-D rendering were compared between the two volumes of interest (VOIs) in the normoxia (D) vs. OIR (E) groups, demonstrating the absence of secondary plexus and vertical sprouts in the OIR mice.** (F)** Immunofluorescence images of flat-mount retinas only captured the phenotype in the primary plexus. **(G)** Quantification of the morphological and topological parameters revealed the statistically significant reduction in total vascular lengths, total volumes, connectivities and branching points in the OIR P12 mice (*p < 0.05, ** *p* < 0.01 *** *p* < 0.001, by unpaired two-tailed Student's t-test, n = 5 per group). Scale bar: 500 µm for A-C; 100 µm for D-E, 500 µm for F.

In response to hyperoxia, the 3-D vascular network (Figure 4A-B, Movies S3-4) and filament tracing results (Figure 4C, Movie S5) in the OIR group were markedly different from the normoxia group (Figure 4). A preferential depletion of the capillaries in the primary plexuses developed in the central region, whereas depletion of vertical sprouts and secondary plexuses occurred in all three regions as evidenced by the selected VOIs (Figure 4D-E). Importantly, the immunofluorescence 2-D flat mount images for both normoxia and OIR groups demonstrated the expected phenotype of central vaso-obliteration only for the primary plexus, but could not distinguish vertical sprouts and secondary plexus changes unlike the LSFM images (Figure 4F). We further compared the topological parameters, including total length (normoxia: 1.2×10^6^ ± 3.1×10^4^; OIR: 2.6×10^5^ ± 1.3×10^4^ µm, *p* < 0.001, n = 5/group), total volume (normoxia: 7.3×10^7^ ±1.4×10^7^; OIR: 3.2×10^7^ ± 8.2×10^6^ µm^3^,* p* < 0.05, n = 5/group), branching points (normoxia: 2.7×10^4^ ± 9.6×10^2^; OIR: 5.8×10^3^ ± 1.3×10^3^,* p* < 0.001, n = 5/group) and connectivity (normoxia: 1.1×10^4^ ± 6.9×10^2^; OIR: 2.3×10^3^ ± 9.1×10^2^,* p* < 0.01, n = 5/group) for the conformationally-intact retinal vascular network (Figure 4G). In summary, our topological analysis allowed for the demonstration and quantification of the preferential obliteration of the outer layer (secondary plexuses) and vertical sprouts of the capillary vasculature under hyperoxic conditions.

### Frequency of clustering coefficients to quantify differences in the local vs. global vascular network

**Figure 5:**
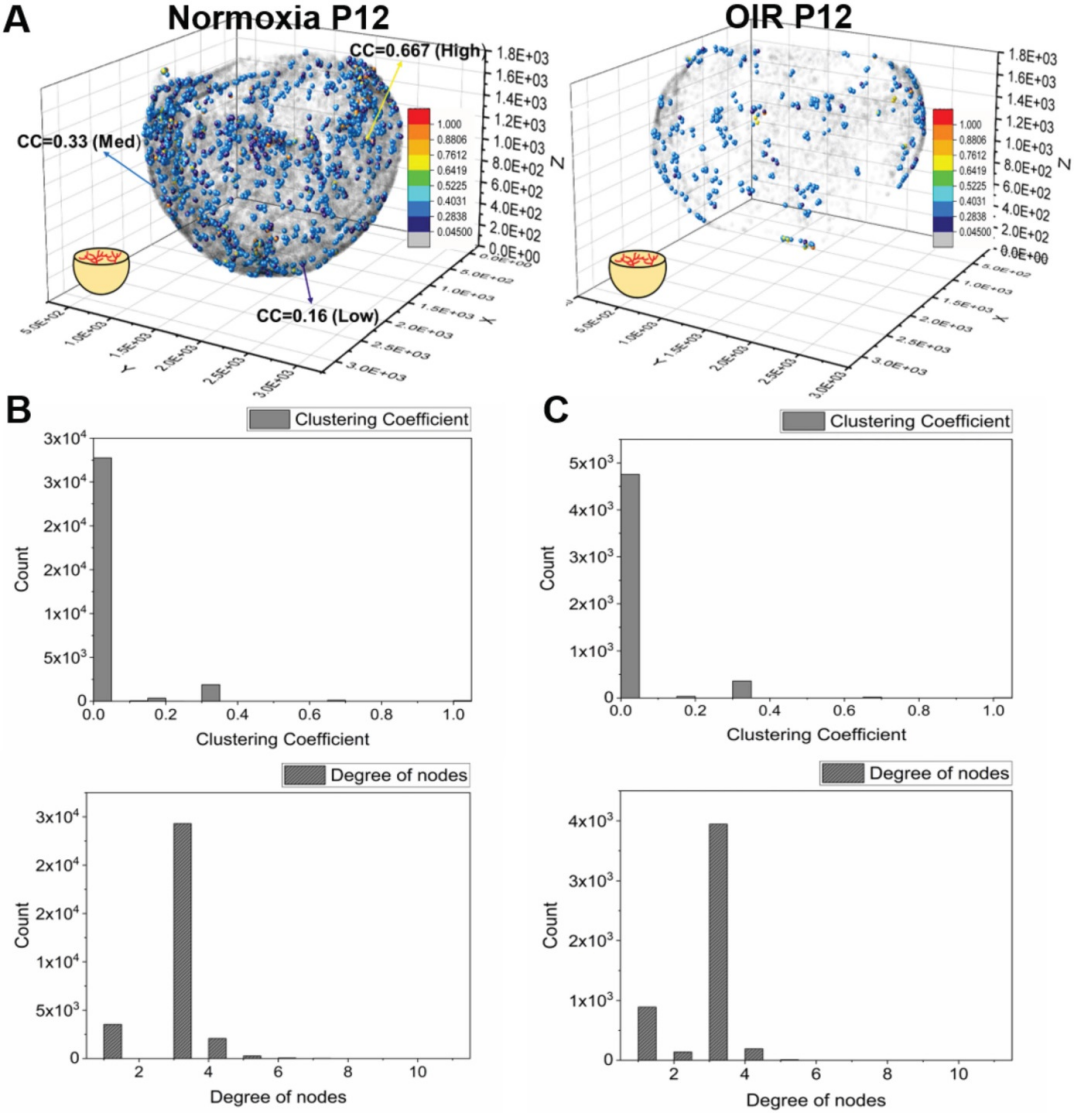
** Quantification of the clustering coefficients for the retinal vasculature in P12 mice under normoxia and OIR conditions. (A)** Representative 3-D scatter plots of the clustering coefficients demonstrate all the branching nodes and terminal nodes traced. ~90% of the nodes have a cluster coefficient of zero (grey nodes). ~10% of the nodes have clustering coefficients ranging from 0.05 to 1 in both normoxia and OIR mice. **(B)** The histogram of clustering coefficients and degree of nodes demonstrates that the majority of the nodes have three connecting neighbors with a paucity of neighbor connection in a normoxia mouse. **(C)** Despite the significant reduction of the global connectivity and number of nodes, the OIR mouse demonstrated the same trend in local patterns as the normoxia mouse, with the majority of the nodes sharing three connecting neighbors with a paucity of neighboring connections.

**Figure 6:**
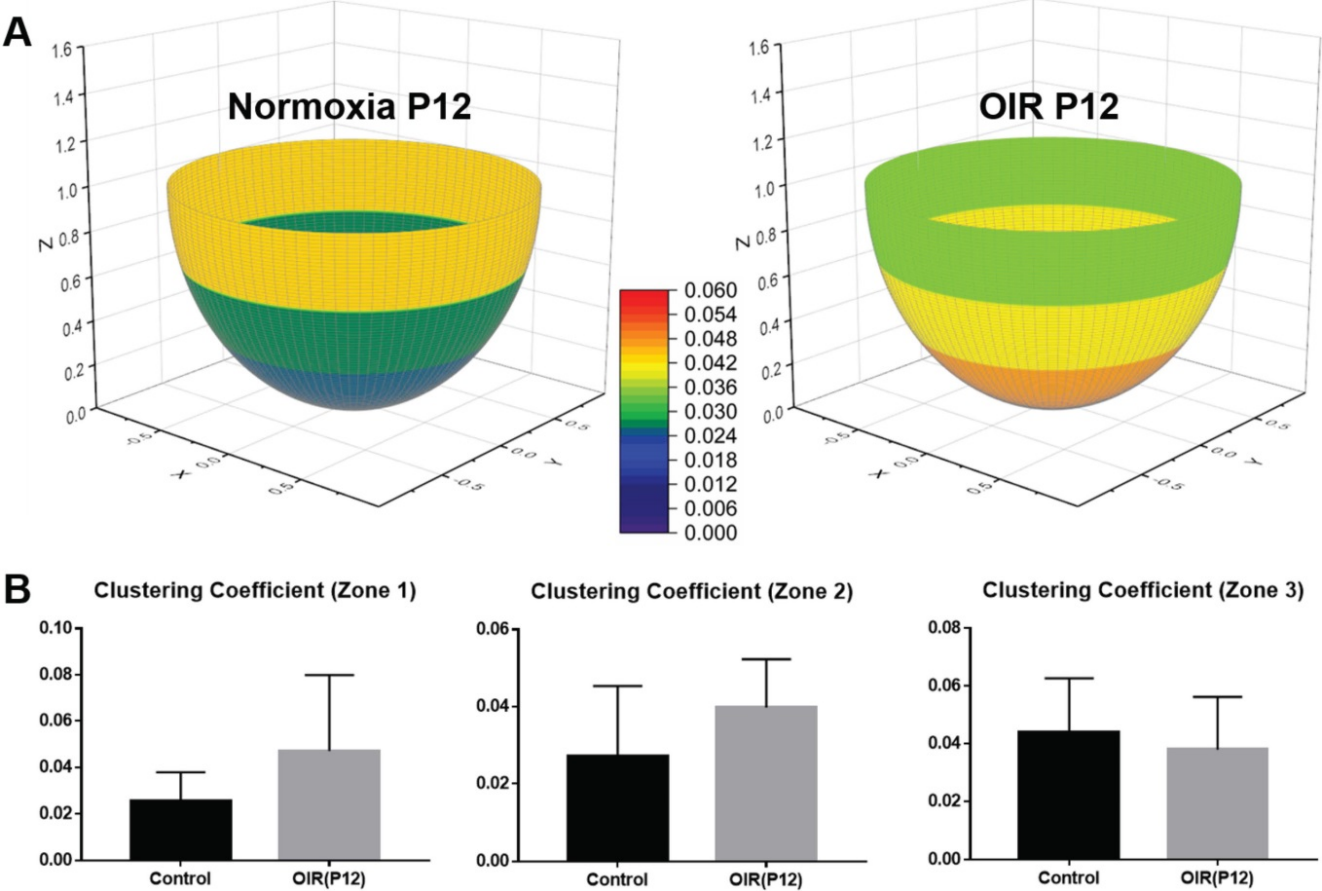
** Average clustering coefficients in different regions of the retina in P12 normoxia and OIR mice. (A)** The retinas were divided into three regions (central, middle, peripheral) as previously described. The average clustering coefficients of three individual regions were calculated and compared between the two groups. **(B)** The average clustering coefficients indicate no statistically significant difference among all three regions in both groups, supporting the notion that the local connections and basic structure of the network are preserved despite vaso-obliteration in OIR retinas (*p*>0.05 for all comparisons by unpaired two-tailed Student's t-test, n = 5 per group).

We computed the distribution of the clustering coefficients for each node in the vascular network (Figure 5A). In both normoxia- and hyperoxia-exposed P12 mice, approximately 10% of the nodes exhibited positive clustering coefficients, whereas 90% of the nodes approached zero (grey), suggesting an overall paucity of connections with the neighboring nodes in the retinal vasculature. The most common clustering coefficients were 0 and the most frequent number of degrees was 3 (Figure 5B-C). These findings demonstrate that most of the nodes form connections with three neighboring nodes, with no connections between its neighbors, resulting in a 3-D reticulated vascular network, analogous to a honeycomb. In addition, the reticulated pattern in the local network was preserved despite hyperoxia-induced obliteration of the capillaries. We further compared the average clustering coefficients among the central, middle, and peripheral regions of the retina (Figure 6A-B), revealing statistically insignificant differences between normoxia and hyperoxia conditions (central = 0.026 ± 0.012 vs. 0.047 ± 0.032, *p* > 0.05; middle = 0.027 ± 0.018 vs. 0.039 ± 0.013, *p* > 0.05; peripheral = 0.044 ± 0.019 vs. 0.038 ± 0.018, *p* > 0.05, n = 5/group). This comparison demonstrated that the local connectivity was preserved, whereas the global connectivity was significantly reduced (local = 0.032 ± 0.018 vs. 0.043 ± 0.024, *p* > 0.05, n = 5/group; global = 1.1×10^4^ ± 6.9×10^2^ vs. 2.3×10^3^ ± 9.1×10^2^, *p* < 0.05, n = 5/group) (Figure 4 and 6). Thus, determination of clustering coefficients supports the preserved local patterning in OIR, but the impaired global capillary connections in response to hyperoxia.

### Topological quantification of the plexuses and vertical sprouts following hyperoxia

**Figure 7:**
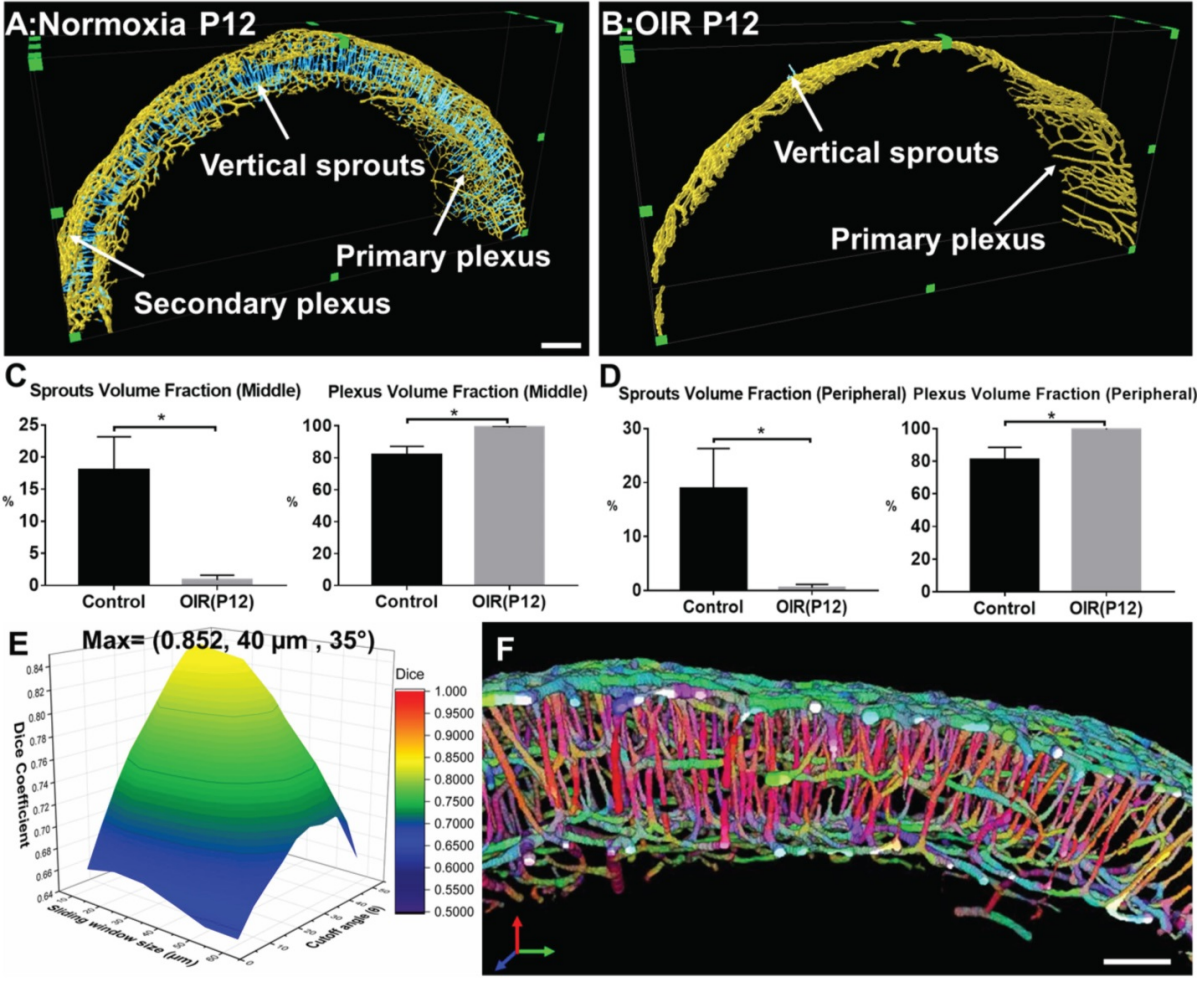
** Quantitative analysis of the volumes for the vertical sprouts and plexuses in P12 normoxia and OIR mice. (A-B)** Severe vaso-obliteration and depletion of vertical sprouts (in blue) and the secondary plexus (in yellow) were demonstrated in the 3-D rendering of the OIR group.** (C-D)** The quantification of the volume fraction of both sprouts and plexuses from both middle and peripheral regions indicate significant vessel depletion in the OIR group (*p < 0.05 vs. normoxia, by unpaired two-tailed Student's t-test, n = 5 per group). **(E)** The surface plot of the dice coefficients on different sizes of sliding windows and values of cutoff angles.** (F)** The representative VOI demonstrating the orientation color-coded 3-D rendering of the vascular network (X: Blue, Y: Green: Z: Red). Scale bar: 200 µm for A-B and 100 µm for F.

The filament tracing results allowed for auto-segmentation of image stacks to construct a 3-D unscathed retinal vascular network. We randomly selected 5 VOIs at 200 µm in thickness to quantify the volume fraction of vertical sprouts, defined as the volume of vertical sprouts divided by the total volume of the vascular network. The representative images for the maximum intensity projection (MIP) of the automatically segmented plexuses and vertical sprouts were assessed from selected image slices (Figure S1). Furthermore, the 3-D rendering results (Figure 7A-B) of the plexuses (in yellow) and vertical sprouts (in blue) demonstrated statistically significant obliteration of the vertical sprouts following hyperoxia treatment (*p*<0.05 vs. normoxia, n=5) (Figure 7A & B). Spatial variations in obliteration were observed; namely, 17.98% ± 5.2% vs. 0.69% ± 0.75% in the middle, and 18.89% ± 7.43% vs. 0.51% ± 0.62% in the peripheral regions (Figure 7C-D). To validate the auto segmentation method, we segmented the vertical sprouts and plexuses by performing manual and automated labeling. The generalized dice coefficients were obtained to compare the similarity between the manual annotation and automated segmentation under each condition. To optimize automated segmentation, we introduced the sliding window (40 µm x 40 µm x 40 µm) with 20 µm in shifting distance and 35° as the cutoff angle (Figure 7E, Tables S1-S3). The maximum dice coefficient and low standard deviation (0.852 ±0.04) indicate accurate segmentation of the vertical sprouts by the auto-segmentation method (Figure 7E, Tables S1-S3). Finally, the representative VOI demonstrated 3-D rendering of the color-coded (X: Blue, Y: Green: Z: Red) vascular networks in relation to the orientation of the vessels (Figure 7F). Taken together, our computational analyses enable multi-scale interrogation of the 3-D vascular network from the micro- to the macro scale.

## Discussion

Advances in imaging modalities have dramatically enhanced the fundamental understanding and experimental capabilities of microvascular injury and repair models, including retinopathy of prematurity and diabetes. Our integration of 3-D imaging with topological computation demonstrated the ability to quantitatively characterize the morphological changes in 3-D of the entire retinal vascular network in a neonatal mouse model of oxygen-induced retinopathy (OIR). The use of clustering coefficients, Euler's number, and PCA corroborated the reticular vascular network undergoing capillary obliteration, revealing spatial variations in capillary obliteration in the deep plexuses and the bridging vessels in response to hyperoxia. Our light-sheet imaging further revealed a 3-D drop-out phenomenon of the deep plexuses preceding capillary obliteration in the central superficial plexuses, otherwise challenging to detect with the 2-D imaging modalities 16, 31.

In the retina, vascular development is regulated by a host of signaling pathways that are coordinated by environmental cues and cellular metabolic activities 2, 3. Murine retinal vasculature provides a viable platform to investigate genetic and epigenetic effects on vascular injury and regeneration 1, 2, 16. Disturbances to the signaling pathways, including changes in oxygen and nutrient provision, promote pathological vaso-obliteration; result in structural and functional abnormalities during angiogenesis 1, 6, 16. In diabetic retinopathy, hypoxia resulting from a non-perfused capillary bed induces vascular endothelial growth factor (VEGF) expression, thereby, promoting neo-angiogenesis and vascular permeability 7. In retinopathy of prematurity, pathological obliteration of the capillary network can progress to irreversible blindness 3, 6. To this end, 3-D laser light-sheet microscopy allows for imaging of the 3-D hemispherical vascular network, uncovering multi-layer vascular changes, and quantifying epigenetic vs. genetic perturbations. LSFM coupled with our topologic analyses could also be adapted to larger animals and even human tissue samples, with translational potential to identify therapeutic targets 16, 24, 32, 33.

The conventional method used to image retinal vascular structures typically involves flat-mount samples, with the fluorescently-labeled structures detected via confocal or fluorescence microscopy 17, 18, 34-40. Unlike wide-field, two-photon and confocal microscopes, LSFM enables rapid optical sectioning at high acquisition rates 25, 26. Optical sectioning generates a sheet of light, allowing for only a selective plane to be illuminated; thereby, minimizing photobleaching 24. LSFM imaging enables rapid image acquisition of the 3-D mouse retina in ~10 mins for high-throughput studies.

Advances in optical clearing techniques have rendered the tissue or organ systems (including retinas) from different animal models transparent for LSFM imaging 22, 23, 25, 26, 33, 41. These techniques remove lipids from tissues to minimize photon scattering while stabilizing the 3-D structural conformation 42-44 to allow for deep tissue penetration for large samples 42, 45-48. Successful 3-D imaging of murine hyperoxia-induced retinopathy often encounters technical challenges with the fragile developing tissues and small specimen size. Previously, Singh et al. demonstrated vaso-obliteration in the rat model 22. Prahst et al. reported the local 'knotted' morphology and vascular tufts seen during neovascularization 23. In our study, we optimized and coupled light-sheet imaging with detailed topological analyses to interrogate the entire retinal vasculature, focusing on the vaso-obliterative phase in the mouse OIR model. We demonstrated the spatial variations in 3-D microvascular obliteration, namely, the loss of primary (inner) vs. secondary (outer) capillary plexuses in response to oxygen-induced retinopathy (OIR). This integration of light-sheet imaging and topological analyses demonstrated that vaso-obliteration preferentially occurs in the outer layer (secondary plexus) and the vertical sprouts.

In addition to imaging developmental biologic processes and tissue regeneration, other laboratories and our group have demonstrated the capacity of LSFM to investigate ophthalmologic disease in small animal models 22, 23. While adaptive optics scanning laser ophthalmoscopy (AOSLO) performs *in vivo* imaging with high spatial and temporal resolution 49-52, the small field of view (FOV) remains a challenge to capture the entire vascular network. Similarly, optical coherence tomography (OCT) performs *in vivo* imaging of the retinal vasculature with a relatively small FOV 53, 54. Also, motion artifacts from patient movement and eye blinking further hamper 3-D vascular imaging 55. In the case of small animal models, confocal microscopy and two-photon microscopy may provide high spatial resolution or deep penetration into the retina; however, the point scan nature of these two imaging modalities usually requires a prolonged period to image the entire retina (> several hours) as opposed to the rapid scanning time (~10 mins) of LSFM 22, 23. In this context, the integration of 3-D imaging with quantitative topological analysis provides the vascular network phenotype for the entire retina to address the limitations of OCT encountered in imaging small animal models.

In the mouse model of oxygen-induced retinopathy, LSFM imaging, followed by topological analyses, quantifies the 3-D morphological and topological parameters for the entire vascular network. In addition to quantifying vessel lengths, vessel volumes, and branching points, topological computation further quantified the global vs. local vascular connectivity by Euler numbers and clustering coefficients. Unlike conventional estimations of the network connections via branching points, Euler number calculations simultaneously reflect both connectivity and the topological features of a 3-D network 56-58, facilitating comprehensive measurement of the connectivity of a capillary network 18, 59-62. Moreover, the clustering coefficients capture the local connectivity and degree of organization of the regional vascular network for every vertex in the vascular or neuronal network 63-65. Using Euler number and clustering coefficient calculations, we demonstrated that in hyperoxia-induced retinopathy, there was a significant reduction in global connectivity, but the local reticular pattern was preserved. We further applied principal component analysis (PCA) to automatically segment the vertical sprouts, and to quantify the volume fraction in the plexuses and vertical sprouts under normoxia vs. hyperoxia conditions. As a result, we were able to corroborate the differential obliteration of the vertical sprouts and the secondary plexus in OIR. Although this PCA-based segmentation method offered the fidelity to segment the vertical sprouts, it is limited in its use when the sliding windows include multiple vessels in different directions (i.e. bifurcations). While smaller window sizes reduce the probability of including bifurcations, the trade-off is to increase the possibility of miscalculation of vessel orientation. Thus, determining and optimizing the window size is essential to include adequate vessel elements for quantification of vessel orientation and minimization of including vessel bifurcations prior to initiating the analysis. A potential improvement may be achieved by combining PCA-based segmentation with machine learning by applying the image labels of vertical sprouts (determined by using different window sizes) as inputs to train the classifiers for segmentation. Despite the well-defined formulas to calculate Euler numbers and clustering coefficients, uncertainties reside in the accuracy of segmentation of the blood vessels using filament tracing. The use of machine learning-based automated segmentation for blood vessels will further strengthen the capacity to abbreviate the segmentation process and to obviate the need for filament tracing. Taken together, the topological computation analyses quantitatively corroborate the spatial variations of vaso-obliteration, identifying impaired vascular branching and global connectivity, and reduced vessel volumes and lengths in OIR retinas.

In sum, 3-D LSFM enables deep tissue penetration to unravel topological changes in the 3-D retinal vasculature. Integration of LSFM with 3-D topological analysis demonstrates global vaso-obliteration, but preserved local 3-D reticular vascular architecture, supporting the differential vascular obliteration from the outer (secondary) plexuses to the vertical sprouts in hyperoxia-induced retinopathy. Overall, the application of clustering coefficients, Euler's number, and PCA to 3-D LSFM images provides vascular insights into OIR, with translational significance for developing therapeutic interventions to prevent visual impairment.

## Material and Methods

### Ethics statement

All animal studies were performed in compliance with the IACUC protocol approved by the UCLA Office of Animal Research and in accordance with the guidelines set by the National Institutes of Health. Humane care and use of animals were observed to minimize distress and discomfort.

### The modified passive CLARITY for retinal imaging

We optimized the use of CLARITY for fixed retinas as previously described 44, 47. The mice were euthanized at P12 for dissection of the ocular globe. We enucleated the intact ocular globe and then fixed globes in 4% paraformaldehyde (PFA) for 1.5 hours. We then removed the cornea, sclera, lens, and choroid while preserving the retina in its 3-D hemispherical configuration. The 3-D retinas were then immersed in 4% paraformaldehyde overnight (Figure 1D(1)), followed by overnight incubation in a monomer solution (4% Acrylamide, 0.05% Bis-Acrylamide , and 0.25% VA-044 initiator (weight/volume) in PBS) (Figure 1D(2)). Next, samples were placed into a 37 °C water bath for 6 h for hydrogel polymerization (Figure 1D(3)). A degassing nitrogen flush or addition of an oil layer was performed to minimize exposure to oxygen. Next, retinas were incubated in the clearing solution (4% weight/volume sodium dodecyl sulfate (SDS) and 1.25% weight/volume boric acid (pH 8.5) at 37 °C to remove non-transparent lipid contents. (Figure 1D(4)). The retina was rinsed for an additional 24 h in 1X PBS to remove residual SDS, followed by incubation in the refraction index matching solution RIMS (40 g histodenz in 30 ml of 0.02 M PBS with 0.01% neutralized sodium azide (pH to 7.5 with NaOH)) to achieve transparency of the tissue. This method enables the preservation of the 3-D structure of the tissue and maintains the integrity of the retinal vasculature during clearing. Selected P12 retinas (n=3) were collected for direct immunostaining without optical clearing (non-cleared group) for comparison (Figure S2).

### Murine oxygen-induced retinopathy (OIR) model

C57BL/6J mice were acquired from the UCLA Division of Laboratory and Animal Medicine colony. All mice were housed in 12:12 h light-dark cycles. Female pregnant mice were fed *ad libitum* with standard rodent chow diet (Pico Lab Rodent Diet 20, cat#5053, Lab Diet, St. Louis, MO) and water during pregnancy and lactation. Oxygen-induced retinopathy (OIR) was produced using standard published guidelines 17. Pups were designated as P0.5 on the morning that they were delivered. Per protocol recommendations, litters were culled to eight pups. Mothers were randomly assigned to normoxia or hyperoxia (OIR) conditions. To induce OIR, the newborn mice were exposed to 75% oxygen continuously in an airtight chamber (Biospherix Proox model 360, Parish, NY, USA) with their nursing mothers from P7 to P12 before removal to room air, whereas the normoxia group remained in room air (21% oxygen) throughout the postnatal period.

### Immunostaining and imaging of flat-mount retinas

Immunostaining and imaging were performed as previously described 66. Briefly, dissected retinas were washed with phosphate-buffered saline (PBS), blocked with blocking buffer (20% fetal bovine serum (FBS), 2% goat serum, 0.05% bovine serum albumin (BSA), and 1%Triton X-100 in PBS) for 1 h, and stained with Alexa594-isolectin GS-IB4 (Invitrogen, Carlsbad, CA, USA) at 4 °C overnight. Retinas were then incised into four equally-sized quadrants and mounted with ProLong mounting medium (Invitrogen). Images were taken at 4X magnification using an AxioCam CCD digital camera (Carl Zeiss) mounted to an inverted epifluorescence microscope (AxioVert 135; Carl Zeiss).

### Immunostaining for unscathed retinal vasculature

The optically transparent retinas were placed into a blocking buffer Perm/Block (1X PBS + 0.3% Triton-X + 0.2% bovine serum albumin + 5% fetal bovine serum) solution for 1 h at room temperature with gentle shaking. Primary biotinylated GS isolectin B4 (1:50, Vector lab, CA) was used to stain retinal vasculature. Following 2 days of incubation at 4 °C, retinas were washed with PBSTX (1X PBS + 0.3% Triton-X). Streptavidin conjugated with Alexa-488 (1:100, Invitrogen, CA) was utilized to amplify primary-specific fluorescence. The retinas were washed with PBSTX (1X PBS + 0.3% Triton-X) following each step.

### LSFM to image the 3-D retinal vascular network

Our custom-built LSFM was adapted for imaging the 3-D retinal microvasculature as previously reported 24, 25, 67. The detection arm is composed of a stereomicroscope (MVX10, Olympus, Japan) with a 1X magnification objective (NA = 0.25), a scientific CMOS (sCMOS, ORCA-Flash4.0 LT, Hamamatsu, Japan), and a series of filters (Exciter: FF01-390/482/532/640; Emitter: FF01-446/510/581/703; Dichroic: Di01-R405/488/532/635, Semrock, New York, USA). The illumination arm, orthogonal to the detection arm, is composed of the continuous wave laser at 473 nm and 532 nm (LMM-GBV1-PF3-00300-05, Laserglow Technologies, Canada) (Figure S3A). The illumination beam was then reshaped by a relay lens composed of two achromatic doublets with f1= 100 mm and f2 = 30 mm, respectively, followed by the objectives (Nikon, 10X/0.3 Plan Fluor) as the illumination Lens. The lateral and axial resolutions are 5.6 µm and 6.6 µm, respectively with the 4X zoom lens (Figure S3B). The confocal parameter was the effective illumination region (dotted orange line in Figure S3B). For image acquisition, the samples were immersed in the refractive index matching solution (RIMS: RI of 1.46-1.48) with a 1% agarose solution in a Borosilicate glass tube (RI = 1.47, Pyrex 7740, Corning, New York, USA) to reduce refraction and reflection from various interfaces. The glass sample holder was placed in a 3-D-printed opening chamber, made of acrylonitrile butadiene styrene (ABS) (uPrint, Stratasys, USA) and it was filled with 99.5% glycerol (RI = 1.47). We applied single illumination to image the retinal samples within the confocal region. The multiple scans (~3 scans and ~3 mins per each scan) and image stitching module in ImageJ 68 (Tile overlap = 15% and regression threshold = 0.9) were applied to acquire the 3-D imaging stacks for the entire retinas.

### Computational analyses of the morphological and topological parameters of the vascular network

The raw data were preprocessed in ImageJ to remove stationary noise 69, 70 and the background was reduced by rolling ball background subtraction (20 pixels in radius). To provide the axial visualization or the depth of the 3-D vascular network, we applied the depth-coded plugin in ImageJ to enhance visualization of the superficial and deep capillaries. In addition, 3-D rendering and semi-automated filament tracing were performed and processed in Amira 6.1. The results of filament tracing were used to quantify both the morphological (Euler-Poincaré characteristic) and topological parameters (clustering coefficients). The Euler number, χ, of the 3-D object was defined as follows 56, 58:

χ = n_0_ - n_1_ + n_2_ - n_3_, (1)

where n_0,_ n_1,_ n_2,_ and n_3_ are the numbers of vertices (V), edges (E), faces (F), and the individual voxels contained in a 3-D object, respectively. In the setting of increasing connected edges, the Euler number decreases while the network becomes well-connected. We provided an analysis of the changes in Euler number in response to the morphological changes in Figure S4A. The Euler number remains the same when the new connections form a branching structure (first row of Figure S4A). In addition, the Euler number is reduced when a new connection forms loops in a reticular-like structure (second row in Figure S4A), whereas the Euler number is increased when the new connection forms disconnected objects (third row in Figure S4A). Thus, two factors are the main contributors to the Euler number: 1) the number of loops (holes), and 2) the numbers of disconnected objects 16. In this context, we defined the connectivity for the vascular network as 59:

Connectivity=1-χ, (2)

In comparison to the Euler number, the clustering coefficient (C) reflects the local connectivity of individual vertices or nodes in the vasculature 63-65, 71. The clustering coefficient of each vertex and the average clustering coefficient C are defined as follows, respectively:


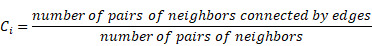



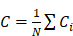
 i=1,2,…N, (3)

The local clustering coefficient *C*_i_ for a vertex v_i_ is given by the proportion of links between the vertices within its neighborhood divided by all possible connections between its neighbors (Figure S4B). For detailed mathematical calculation, a graph G (Equation 4) consists of a set of edges, ***E,*** and vertices, ***V****,* and an edge, e_ij_, connecting vertex v_i_ with vertex v_j_ (Figure S4B). We define the neighborhood set *N*_i_ of vertex v_i_ for its directly connected neighbors (Equation 5). By defining k_i_=|*** N***_i_ | as the number of neighbors in set ***N***_i_ for a vertex v_i_, the clustering coefficient C_i_ for vertex v_i_ can be calculated (Equation 6).

G= (***V***, ***E***), (4)

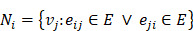
, (5)


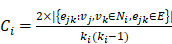
(6)

### Quantitative analysis of vascular plexuses and vertical sprouts in retinal vasculature

The segmented 3D image stacks were derived from the filament tracing results for the retinal vasculature, and they were resampled along the axis passing through and perpendicular to the optical disk. To quantify the volume of vertical sprouts, we developed an automated segmentation method based on principal component analysis (PCA) to reduce the dimensionality for the large data set. Considering a thin section from the retinal vasculature image stack with the origin at the center of mass, the vessels belonging to the primary and secondary plexuses are mainly in azimuthal or polar directions, whereas the majority of the vertical sprouts develop in a radial direction (Figure S1A). To quantify the vessel direction for 3-D image stacks, we introduced a sliding window to sample the principal component representing the vessel orientation inside the window (Figure S1A). The coordinates of each segmented blood vessel inside the sliding window were extracted and recorded for deriving the representative principal component for each window (Figure S1A). Next, we applied the center of the mass as the origin to calculate the angle (θ) between the principal vector of the voxel and the vector from the origin to the center of the voxel (Figure S1A). For validation of the quantification, we utilized the generalized dice coefficient (GDC). It is the general form of the dice similarity coefficient (DSC) which has been widely used to evaluate the accuracy of automated segmentation methods by measuring the similarity of two labels 72, 73. The dice similarity coefficients range from 0 to 1 (DSC>0.7 indicates excellent agreement) and take into consideration the true positive (TP), false positive (FP), and false negative (FN) rates simultaneously 72, 73. The dice similarity coefficient is defined as follows 72:


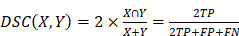
 , (7)

where X represents the manual labeling (ground truth) and Y represents the automated segmentation results. For multiple class segmentation (i.e. vertical sprouts, plexus), the generalized dice coefficient (GDC) is defined as follows 73, 74:


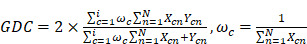
(8)

where N is the number of the image elements, i is the number of classes, and ω is the weighting coefficient. The generalized dice coefficient was calculated for different window sizes and cutoff angles (Figure 7E, Tables S1-S3), and while the vast majority of combinations demonstrated excellent fidelity, with GDCs >0.7, we selected the best combination (GDC of 0.852) to optimize our auto segmentation results.

### Statistical analysis

All data are presented as means ± SD. Statistical significance was determined with unpaired two-tailed Student's t-test for comparison of two groups and one-way ANOVA with Tukey post hoc analysis for multiple group comparisons. The level of significance was set at *p* < 0.05.

## Supplementary Material

Supplementary figures and tables.

Supplementary movie s1.

Supplementary movie s2.

Supplementary movie s3.

Supplementary movie s4.

Supplementary movie s5.

## Abbreviations

ABS: acrylonitrile butadiene styrene
AOSLO: adaptive optics scanning laser ophthalmoscopy
BSA: bovine serum albumin
C: clustering coefficient
DR: diabetic retinopathy
DSC: dice similarity coefficient
E: edges
F: faces
FA: fluorescein angiography
FAZ: foveal avascular zone
FBS: fetal bovine serum
FN: false negative
FOV: field of view
FP: false positive
GDC: generalized dice coefficient
LSFM: light-sheet fluorescence microscopy
MIP: maximum intensity projection
OIR: oxygen-induced retinopathy
OCT: optical coherence tomography
OCTA: optical coherence tomography angiography
PBS: phosphate-buffered saline
PCA: principal component analysis
PFA: paraformaldehyde
P#: postnatal day #
RIMS: refraction index matching solution
ROP: retinopathy of prematurity
SDS: sodium dodecyl sulfate
TP: true positive
V: vertices
VEGF: vascular endothelial growth factor
VOI: volumes of interest
