# Supplementary material for: Three-dimensional Imaging Coupled with Topological Quantification Uncovers Retinal Vascular Plexuses Undergoing Obliteration
Source: Theranostics. 2021 Jan 1;11(3):1162–75. doi: 10.7150/thno.53073 (PMC7738897; doi:10.7150/thno.53073)
Supplement: Supplementary file 1 — Supplementary figures and tables. [file thnov11p1162s1.pdf]

Supplementary Notes

Supplementary movie 1. The 3-D rendering of the vascular network to demonstrate the vertical sprouts embedded between the primary and secondary plexus.

Supplementary movie 2. The filament tracing result of the intact retinal vascular network in normoxia conditions.

Supplementary movie 3. 3-D rendering of the intact retinal vascular network in normoxia conditions.

Supplementary movie 4. 3-D rendering of the abnormal retinal vascular network in OIR.

Supplementary movie 5. The filament tracing of the intact retinal vascular network in OIR.

Representative results for the integration of LSFM and tissue clearing

Representative images of the maximum intensity projection from LSFM were compared between the passive CLARITY-treated retina and control retina, demonstrating the capacity of optical clearing to allow visualization of microvasculature that would otherwise be challenging with conventional imaging modalities (Figure S2A). Note the comparison of pre- and post-CLARITY treatment to allow imaging of the deep vascular plexus (Figure S2B).

Principal component analysis (PCA) of the vasculature

By reducing the dimensions of the data, PCA unravels important characteristics of the data set. It is a linear transformation that generates a new coordinate system from the data such that the greatest variance was introduced to the projected data points to new axes, ordered as components with decreasing variance by the projection of the data [1]. The $\boldsymbol{r}_{i,j}$ (i=1,2…N, j=1-3) represents the coordinate vectors from the vasculature (Equation 1). N represents the numbers of vessel elements inside the sliding window while j represents 3-D space dimensions (X, Y, and Z). The new coordinates are computed from the covariance matrix (Equations 1,2) of the data and applied to the eigenvector decomposition (Equations 3,4) process to derive the new axes known as principal components (PC). The PC1 is the eigenvector with the largest possible

variance and each succeeding component such as PC2 and PC3 has the highest variance possible under the constraint that it is orthogonal to the preceding components. Lastly, the angle between vectors was calculated through the relation of the inner product (Equation 5).

$$Cov(r_j, r_k) = \frac{1}{N}\sum_{i=1}^{N}(r_{i,j} - \overline{r_j})(r_{i,k} - \overline{r_k}) = \sigma_{jk} \qquad \text{(Equation 1)}$$

$$Cov\ Matrix = \begin{bmatrix} \sigma_{xx} & \sigma_{xy} & \sigma_{xz} \\ \sigma_{yx} & \sigma_{yy} & \sigma_{yz} \\ \sigma_{zx} & \sigma_{zy} & \sigma_{zz} \end{bmatrix} \qquad \text{(Equation 2)}$$

Eigen Decomposition

$$Cov\ Matrix = \boldsymbol{V} \cdot \begin{bmatrix} \lambda_1 & 0 & 0 \\ 0 & \lambda_2 & 0 \\ 0 & 0 & \lambda_3 \end{bmatrix} \cdot \boldsymbol{V}^{-1} \qquad \text{(Equation 3)}$$

λ_1 , λ_2 , λ_3 are three eigen values

Eigen Vector ($\boldsymbol{V}$)

$$\boldsymbol{V} = [\overrightarrow{V_1}\ \overrightarrow{V_2}\ \overrightarrow{V_3}] \qquad \text{(Equation 4)}$$

Angle (θ)

$$\theta = \cos^{-1}\frac{\vec{u}\cdot\vec{v}}{|\vec{u}|\cdot|\vec{v}|} \qquad \text{(Equation 5)}$$

1. Jolliffe IT, Cadima J. Principal component analysis: a review and recent developments. Philos Trans A Math Phys Eng Sci. 2016; 374: 20150202.

Supplementary Figure 1

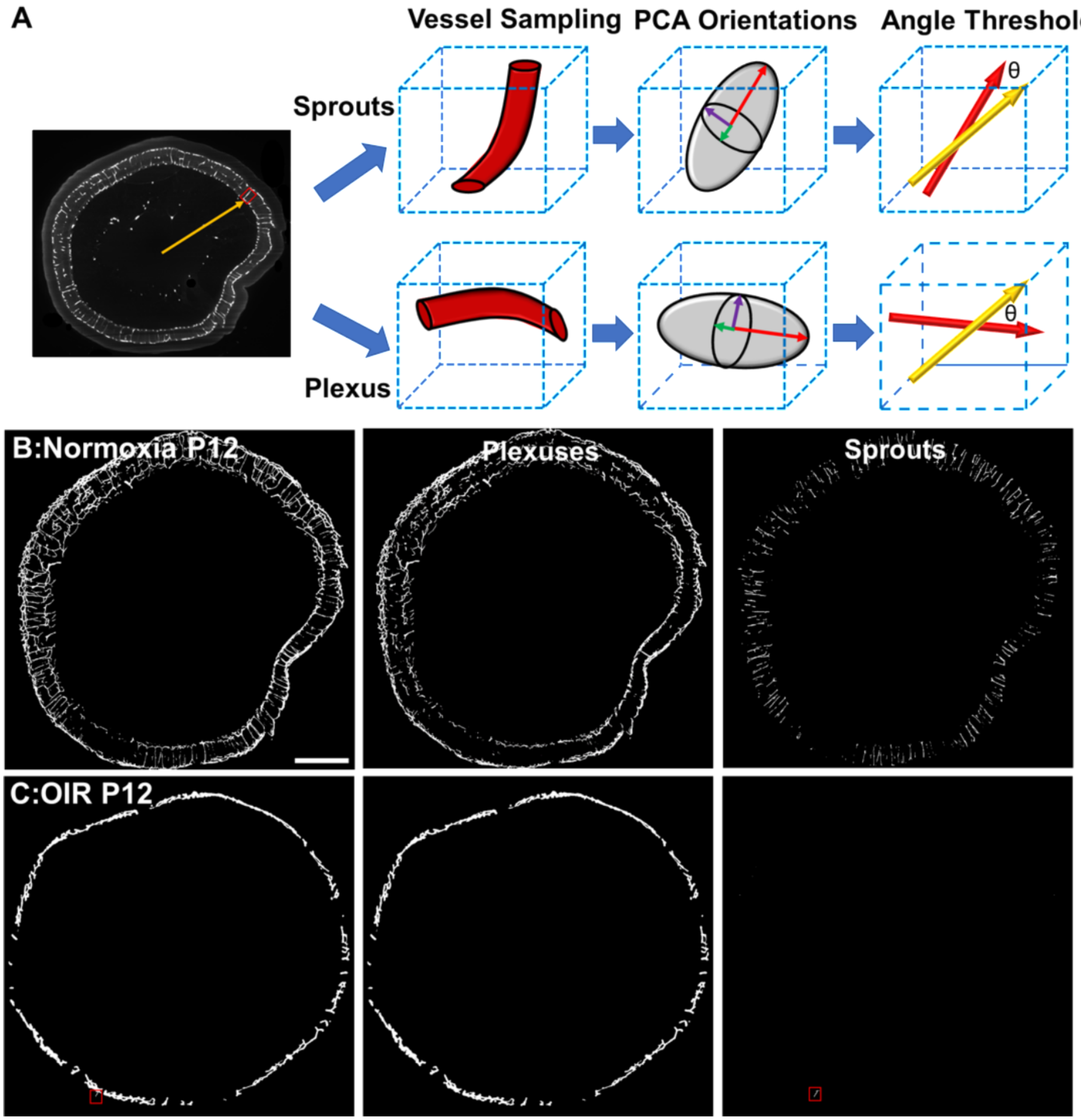

Supplementary Figure 1. Schematic illustration and representative images of the automated segmentation for retinal vertical sprouts and plexuses in P12 mice in normoxia and OIR conditions. (A) The schematic plot indicates the distinct characteristics that distinguish the vertical sprouts and plexuses from each other which serve as the basis for the automated segmentation method. **(B-C)** The representative MIP images from the image stacks demonstrate the successful separation of the vertical sprouts and plexuses using automated segmentation for both normoxia and OIR P12 groups (sprout highlighted in a red box in OIR group). Scale bar: 500 µm.

Supplementary Figure 2

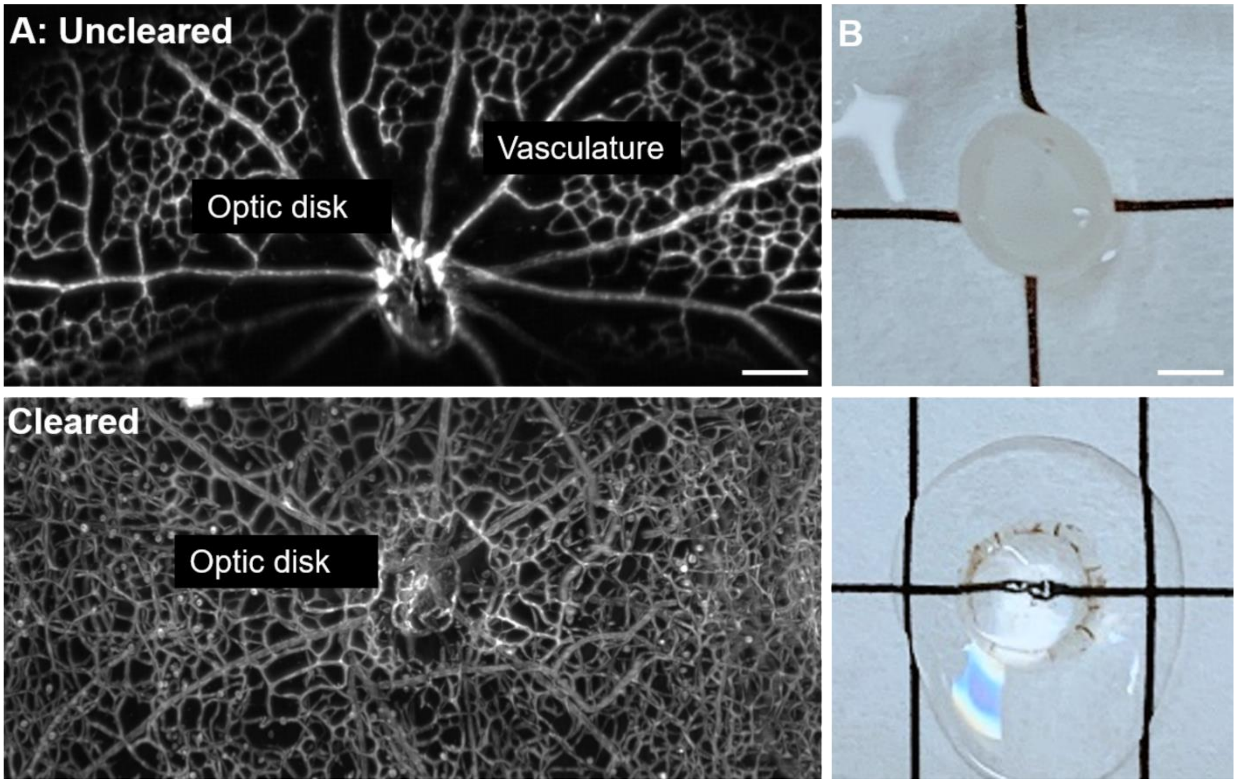

Supplementary Figure 2. Representative images comparing the P12 murine retinas with and without tissue clearing. (A) The detailed vasculature seen after tissue clearing using the CLARITY method is demonstrated using the maximum intensity projection (bottom) in comparison to the uncleared retina (top). **(B)** Gross images of P12 mouse retinas prior to (top) and after (bottom) modified CLARITY treatment. Scale bar: (A) 100 µm; (B) 1 mm.

Supplementary Figure 3

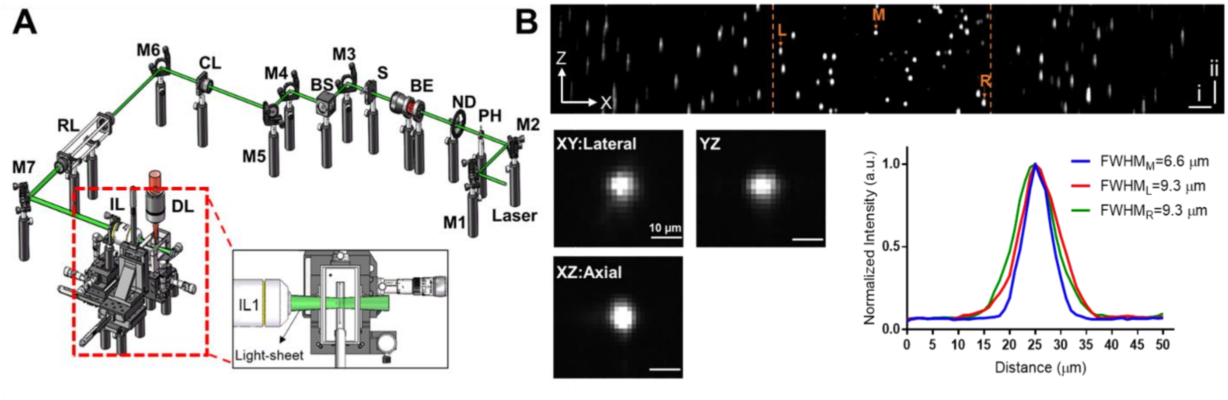

Supplementary Figure 3. A schematic diagram of LSFM. (A) The diagram depicts the individual optical components that are featured in the LSFM system. M1-7: mirror; PH: pinhole; ND: neutral density filter; BE: beam expander; S: slit; BS: beam splitter, CL: cylindrical lens; RL: Relay lens; IL: illumination lens; DL: detection lens. **(B)** Representative images depicting the point spread function (PSF) of the 0.53 µm fluorescent bead in the lateral and axial directions, respectively. The XZ-view provides the characteristics of the confocal zone (bordered by orange dotted lines). Scale bars in panel B represent 100 µm (i-axis) and 50 µm (ii-axis).

Supplementary Figure 4

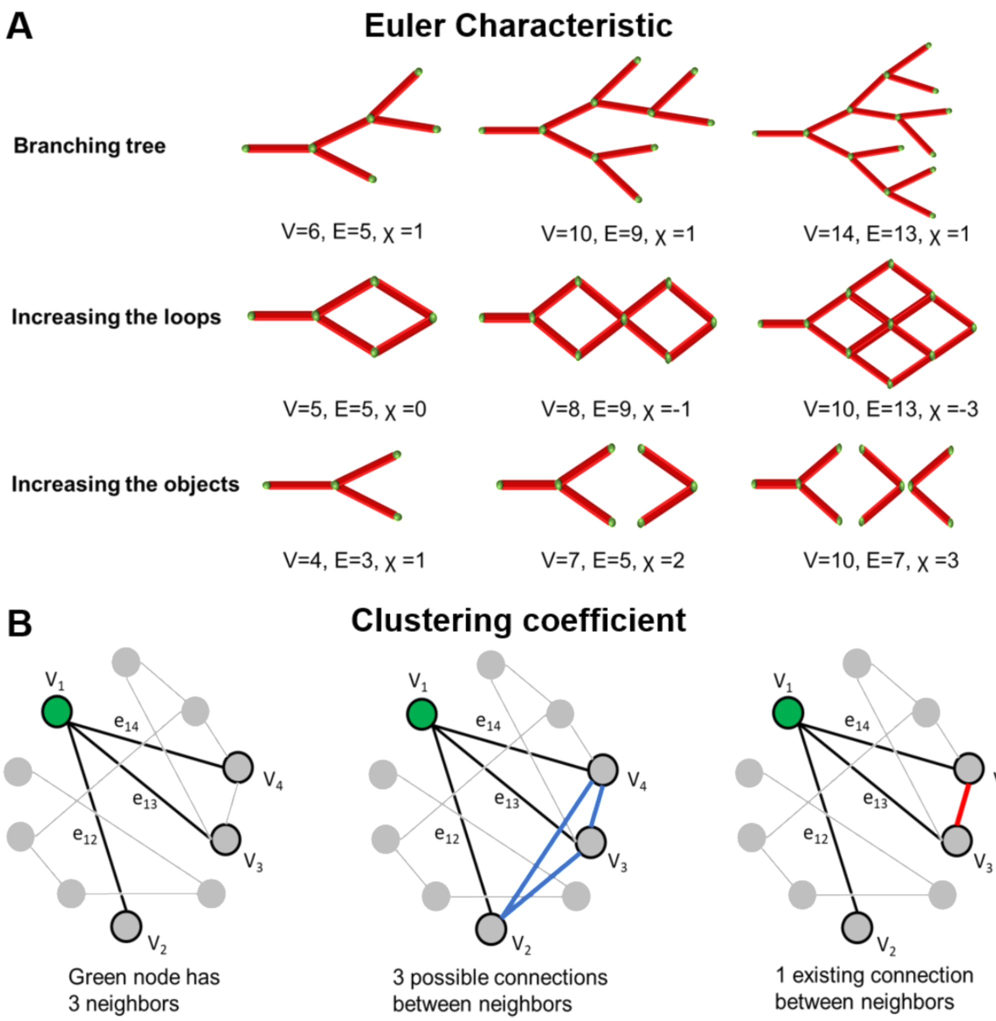

Supplementary Figure 4. Schematic representation to quantify Euler characteristics and clustering coefficients. (A) Euler characteristic, χ, for the vascular network is determined by the numbers of loops and objects in a network rather than by the number of branching points. Both factors (loops and objects) generate new nodes and edges differently and contribute to the difference in the Euler characteristic value. The retinal vascular network is more reticular-like in structure (depicted by the middle row), resulting in a reduction in the χ value. V: Vertices, E: edges. **(B)** The definition of the clustering coefficient for a specific node (green) is calculated by dividing the existing links (red) over all possible links (blue) among neighboring nodes, such that the clustering coefficient of the representative schematic is 0.33.

Dice coefficient for normoxia group

	10 μm	20 μm	30 μm	40 μm	50 μm	60 μm
**5°**	0.66 ± 0.011	0.666 ± 0.015	0.664 ± 0.02	0.655 ± 0.015	0.645 ± 0.01	0.645 ± 0.014
**10°**	0.696 ± 0.026	0.714 ± 0.037	0.707 ± 0.039	0.697 ± 0.034	0.675 ± 0.028	0.666 ± 0.022
**15°**	0.728 ± 0.036	0.755 ± 0.053	0.75 ± 0.056	0.739 ± 0.047	0.712 ± 0.033	0.679 ± 0.039
**20°**	0.756 ± 0.041	0.795 ± 0.055	0.789 ± 0.055	0.775 ± 0.048	0.737 ± 0.039	0.692 ± 0.054
**25°**	0.776 ± 0.043	0.828 ± 0.045	0.822 ± 0.05	0.806 ± 0.039	0.756 ± 0.049	0.707 ± 0.055
**30°**	0.787 ± 0.045	0.845 ± 0.037	0.836 ± 0.041	0.817 ± 0.033	0.77 ± 0.044	0.713 ± 0.052
**35°**	0.793 ± 0.04	0.849 ± 0.032	0.841 ± 0.032	0.822 ± 0.023	0.768 ± 0.042	0.71 ± 0.049
**40°**	0.792 ± 0.035	0.841 ± 0.022	0.836 ± 0.017	0.807 ± 0.015	0.763 ± 0.04	0.712 ± 0.04
**45°**	0.784 ± 0.03	0.825 ± 0.014	0.817 ± 0.009	0.784 ± 0.014	0.749 ± 0.029	0.703 ± 0.036
**50°**	0.768 ± 0.028	0.789 ± 0.022	0.776 ± 0.026	0.75 ± 0.03	0.714 ± 0.023	0.664 ± 0.046

Supplementary Table 1. Average dice coefficients of various combinations of sliding window size and cutoff angle in the normoxia group.

Dice coefficient for OIR group

	10 μm	20 μm	30 μm	40 μm	50 μm	60 μm
**5°**	0.865 ± 0.04	0.868 ± 0.04	0.873 ± 0.037	0.874 ± 0.036	0.874 ± 0.036	0.874 ± 0.036
**10°**	0.842 ± 0.062	0.851 ± 0.058	0.872 ± 0.035	0.879 ± 0.034	0.887 ± 0.036	0.877 ± 0.035
**15°**	0.821 ± 0.072	0.839 ± 0.062	0.894 ± 0.053	0.899 ± 0.049	0.886 ± 0.037	0.9 ± 0.044
**20°**	0.807 ± 0.07	0.828 ± 0.057	0.864 ± 0.077	0.898 ± 0.049	0.883 ± 0.036	0.884 ± 0.032
**25°**	0.796 ± 0.076	0.82 ± 0.064	0.864 ± 0.065	0.896 ± 0.052	0.907 ± 0.047	0.881 ± 0.029
**30°**	0.777 ± 0.081	0.813 ± 0.068	0.855 ± 0.061	0.882 ± 0.045	0.892 ± 0.041	0.88 ± 0.029
**35°**	0.757 ± 0.089	0.8 ± 0.071	0.848 ± 0.064	0.882 ± 0.029	0.889 ± 0.038	0.89 ± 0.016
**40°**	0.739 ± 0.094	0.782 ± 0.071	0.834 ± 0.066	0.882 ± 0.027	0.874 ± 0.06	0.892 ± 0.014
**45°**	0.72 ± 0.1	0.767 ± 0.08	0.826 ± 0.07	0.874 ± 0.027	0.871 ± 0.061	0.883 ± 0.02
**50°**	0.7 ± 0.103	0.74 ± 0.092	0.793 ± 0.102	0.799 ± 0.096	0.794 ± 0.143	0.798 ± 0.106

Supplementary Table 2. Average dice coefficients of various combinations of sliding window size and cutoff angle in the OIR group.

Dice coefficient for both groups

	10 μm	20 μm	30 μm	40 μm	50 μm	60 μm
**5°**	0.763 ± 0.113	0.767 ± 0.111	0.769 ± 0.115	0.765 ± 0.12	0.76 ± 0.125	0.76 ± 0.125
**10°**	0.769 ± 0.088	0.783 ± 0.085	0.789 ± 0.095	0.788 ± 0.102	0.781 ± 0.117	0.771 ± 0.116
**15°**	0.775 ± 0.069	0.797 ± 0.068	0.822 ± 0.092	0.819 ± 0.096	0.799 ± 0.098	0.79 ± 0.124
**20°**	0.782 ± 0.057	0.811 ± 0.054	0.826 ± 0.071	0.836 ± 0.079	0.81 ± 0.085	0.788 ± 0.111
**25°**	0.786 ± 0.054	0.824 ± 0.049	0.843 ± 0.056	0.851 ± 0.063	0.831 ± 0.092	0.794 ± 0.102
**30°**	0.782 ± 0.057	0.829 ± 0.05	0.846 ± 0.047	0.849 ± 0.049	0.831 ± 0.076	0.796 ± 0.098
**35°**	0.775 ± 0.062	0.824 ± 0.054	0.845 ± 0.044	0.852 ± 0.04	0.829 ± 0.074	0.8 ± 0.103
**40°**	0.765 ± 0.066	0.812 ± 0.054	0.835 ± 0.04	0.844 ± 0.044	0.819 ± 0.074	0.802 ± 0.1
**45°**	0.752 ± 0.07	0.796 ± 0.056	0.821 ± 0.041	0.829 ± 0.052	0.81 ± 0.077	0.793 ± 0.101
**50°**	0.734 ± 0.073	0.764 ± 0.061	0.784 ± 0.063	0.775 ± 0.065	0.754 ± 0.094	0.731 ± 0.1

Supplementary Table 3. Average dice coefficients of various combinations of sliding window size and cutoff angle in both groups combined.
